# Chemoresistance acquisition induces a global shift of expression of aniogenesis-associated genes and increased pro-angogenic activity in neuroblastoma cells

**DOI:** 10.1186/1476-4598-8-80

**Published:** 2009-09-29

**Authors:** Martin Michaelis, Denise Klassert, Susanne Barth, Tatyana Suhan, Rainer Breitling, Bernd Mayer, Nora Hinsch, Hans W Doerr, Jaroslav Cinatl, Jindrich Cinatl

**Affiliations:** 1 Institut für Medizinische Virologie, Klinikum der J.W. Goethe-Universität, Paul Ehrlich-Str. 40, 60596 Frankfurt am Main, Germany; 2 blue-drugs GmbH, Komturstr. 3A, 60528 Frankfurt am Main, Germany; 3 Groningen Bioinformatics Centre, University of Groningen, Kerklaan 30, 9751 NN Haren, The Netherlands; 4 emergentec biodevelopment GmbH, Rathausstr. 5/3, 1010 Vienna, Austria; 5 Senckenbergisches Institut für Pathologie, Klinikum der J.W. Goethe Universität, Theodor Stern-Kai 7, 60590 Frankfurt am Main, Germany

## Abstract

**Background:**

Chemoresistance acquisition may influence cancer cell biology. Here, bioinformatics analysis of gene expression data was used to identify chemoresistance-associated changes in neuroblastoma biology.

**Results:**

Bioinformatics analysis of gene expression data revealed that expression of angiogenesis-associated genes significantly differs between chemosensitive and chemoresistant neuroblastoma cells. A subsequent systematic analysis of a panel of 14 chemosensitive and chemoresistant neuroblastoma cell lines in vitro and in animal experiments indicated a consistent shift to a more pro-angiogenic phenotype in chemoresistant neuroblastoma cells. The molecular mechanims underlying increased pro-angiogenic activity of neuroblastoma cells are individual and differ between the investigated chemoresistant cell lines. Treatment of animals carrying doxorubicin-resistant neuroblastoma xenografts with doxorubicin, a cytotoxic drug known to exert anti-angiogenic activity, resulted in decreased tumour vessel formation and growth indicating chemoresistance-associated enhanced pro-angiogenic activity to be relevant for tumour progression and to represent a potential therapeutic target.

**Conclusion:**

A bioinformatics approach allowed to identify a relevant chemoresistance-associated shift in neuroblastoma cell biology. The chemoresistance-associated enhanced pro-angiogenic activity observed in neuroblastoma cells is relevant for tumour progression and represents a potential therapeutic target.

## Background

Neuroblastoma is the most frequent extracranial solid tumour of childhood. About half of all neuroblastoma patients are diagnosed with high-risk disease with overall survival rates below 40% despite intensive multimodal treatment [1]. Therapy failure is basically caused by acquired chemoresistance. Primary tumours usually respond to initial chemotherapy. However, a significant fraction of tumours reappear as chemoresistant recidives [2].

Acquisition of chemoresistance under therapy may affect the biology of neuroblastoma and other tumour cells [3-9]. Mostly a shift towards a more malignant phenotype is observed indicating cancer progression [3,4,6-9]. Molecular changes in different signalling pathways including apoptosis signalling or cell cycle regulation may be involved in this coincidence of cancer cell chemoresistance and increased malignancy [3,10,11]. Neuroblastoma cells adapted to different cytotoxic drugs showed increased malignant properties as indicated by enhanced invasive potential in vitro [7,8] and increased malignancy in nude mice [9].

Here, differences in angiogenesis signalling were identified by bioinformatics pathway analysis of gene expression data from chemosensitive and chemoresistant neuroblastoma cells. Subsequently, cell culture and animal experiments using 14 human neuroblastoma cell lines indicated a consistently higher pro-angiogenic activity of chemoresistant neuroblastoma cells than of chemosensitive cells. The molecular mechanisms underlying the chemoresistance-associated increased pro-angiogenic potential were individual and differed between individual cell lines. Doxorubicin treatment of doxorubicin-resistant neuroblastoma xenografts resulted in impairment of tumour angiogenesis and growth suggesting the chemoresistance-associated pro-angiogenic phenotype to contribute to tumour progression.

## Methods

### Gene expression analysis

Gene expression analysis using AB1700 Human Genome Survey Microarray V2.0 chips (Applied Biosystems, Darmstadt, Germany) was performed by IMGM laboratories (Martinsried, Germany). Gene expression analysis using GeneChip HGU133 Plus 2.0 (Affymetrix, Santa Clara, CA, USA) was performed by Fraunhofer Institut für Zelltherapie und Immunologie (Leipzig, Germany). mRNA was isolated using the RNeasy kit (Qiagen, Hilden, Germany) according to the manufacturer's instructions. Triplicates of UKF-NB-3 RNA were compared to triplicates of UKF-NB-3^r^VCR^10 ^RNA, UKF-NB-3^r^DOX^20 ^RNA, or UKF-NB-3^r^CDDP^1000 ^RNA.

AB1700 expression data were processed using the R/bioconductor package '*ABarray*' (; ) with default parameter (SN threshold > = 3, % Detect Samples: 0.5). This included quality control, quantile normalisation [12] and filtering of unspecific hybridisation. HGU133 Plus 2.0 expression data were processed using the R/bioconductor packages '*gcrma*' and '*limma*' (; ).

For every microarray experiment, the expression pattern of 50 randomly chosen genes was verified by quantitative real-time PCR resulting in confirmation of expression of >80% of investigated genes (data not shown).

### Signal transduction pathway bioinformatics

Statistical analysis to identify significant expression changes was focusing on a pathway analysis using the PANTHER database [13], which identifies global patterns in expression. For each expert-curated pathway in the database, potential differential expression was determined by a binomial test [14], using the PANTHER human gene reference list matching our microarrays (Human AB1700 genes) and lists of differentially expressed genes that passed a false discovery rate threshold [15] of 0.05 based on a t-test.

A total of 25,909 genes were annotated in the dataset, 3,125 of them included pathway information, and 223 of these (corresponding to 280 AB1700 ProbeIDs or 537 HGU133 Plus 2.0 ProbeIDs) were annotated as related to angiogenesis. For this list of angiogenesis-associated ProbeIDs and angiogenesis-associated genes signal intensities of UKF-NB-3, UKF-NB-3^r^VCR^10^, UKF-NB-3^r^CDDP^1000^, or UKF-NB-3^r^DOX^20 ^cells were visualised as heatmaps using R .

### Cells

The cell lines UKF-NB-2, UKF-NB-3, and UKF-NB-4 were isolated from bone marrow metastases from N-myc-amplified stage 4 neuroblastoma patients [16-18]. Be(2)-C cells and IMR-32 cells were obtained from ATCC (Manassass, VA, USA). Be(2)-C cells and UKF-NB-4 cells were isolated as chemoresistant cell lines from patients [17]. The parental UKF-NB-2, UKF-NB-3 or IMR-32 cells are chemosensitive (no P-gp expression, wild-type p53) [19]. Cells were adapted to growth in the presence of vincistine (10 ng/ml), doxorubicin (20 ng/ml), or cisplatin (1000 ng/ml) as described and named following the published nomenclature [16-20], e.g. UKF-NB-3^r^VCR^10 ^means UKF-NB-3 adapted to vincristine 10 ng/ml, UKF-NB-3^r^DOX^20 ^means UKF-NB-3 adapted to doxorubicin 20 ng/ml, UKF-NB-3^r^CDDP^1000 ^means UKF-NB-3 adapted to cisplatin 1000 ng/ml. P-glycoprotein (P-gp) expression and p53 status are shown in Table 1.

**Table 1:** Influence of supernatants from different neuroblastoma cell lines on endothelial cell growth and endothelial cell survival

	**P-gp^1^**	**p53 mutation^2^**	**Endothelial cell growth (%)^3^**	**Endothelial cell survival (%)^4^**
Positive control^5^			100 ± 8	100 ± 9
Negative control^6^			5 ± 7	12 ± 6
UKF-NB-3	-	-	15 ± 6	22 ± 8
UKF-NB-3^r^VCR^10^	+	+ (C135F)	78 ± 12*	83 ± 12*
UKF-NB-3^r^CDDP^1000^	-	-	89 ± 9*	85 ± 7*
UKF-NB-3^r^DOX^20^	+	-	66 ± 13*	70 ± 6*
UKF-NB-2	-	-	17 ± 11	20 ± 5
UKF-NB-2^r^VCR^10^	+	-	48 ± 13*	55 ± 8*
UKF-NB-2^r^CDDP^1000^	-	-	38 ± 7*	48 ± 10*
UKF-NB-2^r^DOX^20^	+	-	35 ± 9*	42 ± 9*
IMR-32	-	-	8 ± 6	17 ± 5
IMR-32^r^VCR^10^	+	-	63 ± 15*	67 ± 8*
IMR-32^r^CDDP^1000^	-	-	49 ± 7*	58 ± 6*
IMR-32^r^DOX^20^	-	-	37 ± 8*	44 ± 14*
UKF-NB-4	+	+ (C175F)	92 ± 9^#^	78 ± 15^#^
Be(2)-C	+	+ (C135F)	94 ± 16^#^	90 ± 7^#^

^1 ^P-glycoprotein (P-gp) expression; + = overexpression, - = no overexpression

^2 ^p53 status; + = mutated p53, - = wild-type p53

^3 ^endothelial cells were grown in cancer cell culture supernatants mixed 1:1 with IMDM supplemented with 10% FCS; cell number was determined after 120 h

^4 ^confluent endothelial cell monolayers were incubated with cancer cell culture supernatants mixed 1:1 with IMDM supplemented with 10% FCS; cell number was determined after 48 h

^5 ^IMDM supplemented with 15% FCS, 5% human serum, bFGF 2.5 ng/ml

^6 ^IMDM supplemented with 10% FCS

* P < 0.05 relative to corresponding parental chemosensitive neuroblastoma cell line

^# ^P < 0.05 relative to the parental chmosensitive neuroblastoma cell lines UKF-NB-3, UKF-NB-2, and IMR-32.

All cell lines were grown in Iscove's modified Dulbecco's medum (IMDM) supplemented with 10% foetal calf serum (FCS), 100 IU/ml penicillin, and 100 mg/ml streptomycin at 37°C.

Human umbilical vein endothelial cells (HUVECs) were cultivated as described before [21] using IMDM supplemented with 15% FCS and 5% pooled human serum.

### Viability assay

HUVEC viability was investigated using the CellTiter-Glo^® ^Luminescent Cell Viability Assay (Promega, Mannheim, Germany) following the manufacturer's instructions.

### Caspase activation

Caspase 3/7 activation was measured using the Caspase-Glo 3/7 Assay (Promega, Mannheim, Germany) following the manufacturer's instructions.

### Tube formation assay

Endothelial cellular tube formation was investigated using HUVECs seeded on extracellular matrix (Matrigel, BD Biosciences, Heidelberg, Germany) as described before [21].

### Western blot

Cells were lysed in Triton X-sample buffer and separated by SDS-PAGE. Proteins were detected using specific antibodies against β-actin (Sigma, Taufkirchen, Germany), ERK 1/2, the phosphorylated forms of ERK 1/2 (each from New England Biolabs, Frankfurt am Main, Germany), Akt, or the phosphorylated forms of Akt (all Millipore (Upstate), Schwalbach, Germany) and were visualised by enhanced chemiluminescence using a commercially available kit (Amersham, Freiburg, Germany).

### Electrophoretic mobility shift assay (EMSA)

Electrophoretic mobility shift assay (EMSA) was performed as described [22].

### Animal experiments

Experiments using the chick chorioallantoic membrane (CAM) were performed using described methods [23]. 10^6 ^cells were placed onto the CAM at day 8. Vessel formation was examined at day 12.

Mouse experiments were performed using female NMRI:nu/nu mice as described before [9]. 10^7 ^cells were injected subcutaneously together with Matrigel in a total volume of 100 μl. For doxorubicin treatment, the day when xenograft tumours became palpable was defined to be day 1. Tumour sections were stained for apoptotic cells by TUNEL staining and for cell proliferation by ki67 staining using established methods [24,25].

All animal experiments were performed in accordance with all relevant declarations on the use of laboratory animals and with the German Animal Protection Law.

## Results

### Expression of angiogenesis-associated genes

A pathway analysis was performed in order to detect the most strongly influenced signalling pathways between UKF-NB-3 and its chemoresistant sub-lines UKF-NB-3^r^VCR^10 ^and UKF-NB-3^r^CDDP^1000^. Of the 153 pathways mapped at PANTHER, angiogenesis was found to be the fourth most significantly affected signalling pathway (p = 1.87 × 10^-4^) [see Additional file 1].

**Figure 1:**
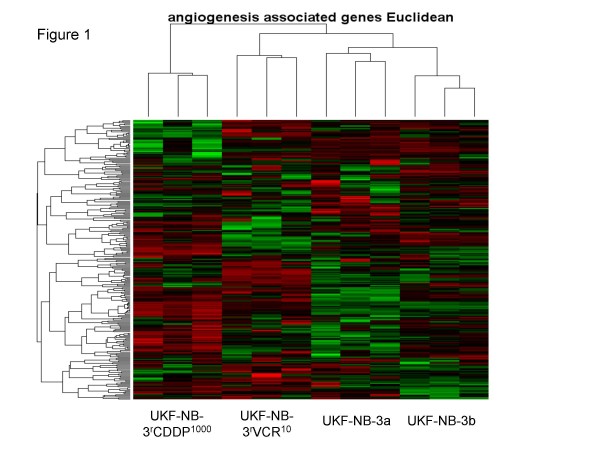
**Hierarchical cluster analysis and heatmap showing expression of angiogenesis-associatd genes (taken from PANTHER pathway) in UKF-NB-3, UKF-NB-3^r^VCR^10^, or UKF-NB-3^r^CDDP^1000 ^cells**. Data were merged from two independent exeriments each comparing UKF-NB-3 with one chemoresistant cell line. UKF-NB-3a was analysed together with UKF-NB-3^r^VCR^10^, UKF-B-3b was analysed together with UKF-NB-3^r^CDDP^1000^.

Hierarchical cluster analysis and the heatmap indicating expression of angiogenesis-associated ProbeIDs (Figure 1) illustrate a striking and consistent re-arrangement of angiogenesis-related gene expression in the resistant cells. The 39 angiogenesis-related ProbeIDs differentially regulated between UKF-NB-3 and UKF-NB-3^r^VCR^10 ^cells represent 35 genes [see Additional file 2]. Of these 35 genes, 27 were up-regulated in UKF-NB-3^r^VCR^10 ^cells in comparison to UKF-NB-3 cells and 8 were down-regulated. The 10 angiogenesis-related ProbeIDs differentially regulated between UKF-NB-3 and UKF-NB-3^r^CDDP^1000 ^cells represent 10 genes [see Additional file 3]. Of these 10 genes, 8 were up-regulated in UKF-NB-3^r^CDDP^1000 ^cells in comparison to UKF-NB-3 cells and 2 were down-regulated.

Subsequently to these analyses, we compared angiogenesis signalling between UKF-NB-3 and UKF-NB-3^r^DOX^20 ^cells. Since Applied Biosystems had stopped manufacturing of AB1700 arrays, HGU133 Plus 2.0 arrays (Affymetrix) were used. Results were similar to those obtained from the comparison of UKF-NB-3 with UKF-NB-3^r^VCR^10 ^and UKF-NB-3^r^CDDP^1000 ^cells. PANTHER pathway analysis indicated angiogenesis to be the fourth most significantly differentially regulated signalling pathway [see Additional file 4]. Hierarchical cluster analysis of angiogenesis-associated genes separated UKF-NB-3 from UKF-NB-3^r^DOX^20 ^cells [see Additional file 5]. 65 angiogenesis-associated genes were found significantly differentially regulated between UKF-NB-3 and UKF-NB-3^r^DOX^20 ^cells. 38 genes were up-regulated in UKF-NB-3^r^DOX^20 ^cells and 27 genes were down-regulated in UKF-NB-3^r^DOX^20 ^relative to UKF-NB-3 cells [see Additional file 6]. The relatively high number of significantly differentially regulated genes compared to the comparisons of UKF-NB-3 vers. UKF-NB-3^r^VCR^10 ^or UKF-NB-3^r^CDDP^1000 ^cells most likely results from the different statistical procedures used to analyse HGU133 Plus 2.0 or AB1700 data.

To further investigate the influence of chemoresistance acquisition on the pro-angiogenic potential of cancer cells, a panel of chemsensitive and chemoresistant neuroblastoma cell lines was systematically investigated for their angiogenic phenotypes.

### Influence of neuroblastoma cell line supernatants on endothelial cell growth and survival

**Figure 2:**
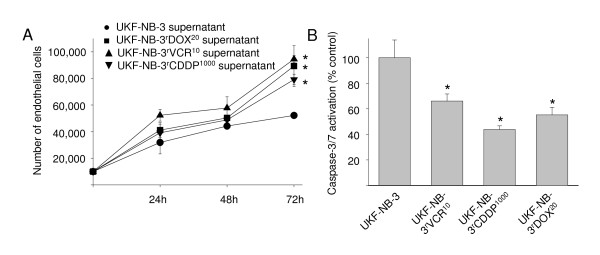
**Influence of neuroblastoma cell culture supernatants on endothelial cell growth and viability**. A) Cell growth characteristics of human umbilical vein endothelial cells (HUVECs) incubated with a mixtures of neuroblastoma cell culture supernatants and IMDM (1:1) supplemented with 10% FCS indicated by cell count. B) Caspase 3/7 activation in confluent endothelial cell monolayers incubated with a mixtures of neuroblastoma cell culture supernatants and IMDM (1:1) supplemented with 10% FCS for 48 h. * P < 0.05 relative to endothelial cells incubated with UKF-NB-3 supernatants.

Neuroblastoma cell lines were grown for seven days. Then medium was removed, cells were washed and protein-free medium was added. After 48 h incubation, supernatants were collected, adjusted to the same protein content, mixed in a 1:1 ratio with fresh IMDM, and FCS (resulting in 10% FCS) was added (unless otherwise noted, all cell culture supernatants were handled following this procedure). HUVECs were trypsinised and suspended in the mixtures of supernatants, fresh IMDM and FCS (without further addition of growth factors). 10^3 ^cells suspended in 100 μl of respective medium were seeded per well in 96-well plates. After five days, HUVEC growth was examined by viability assay (Table 1). HUVECs suspended in IMDM plus 10% FCS did not grow (negative control). HUVECs suspended in IMDM plus 15% FCS, 5% pooled human serum, and basic fibroblast growth factor (bFGF) 2.5 ng/ml formed vital, closely grown monolayers (positive control). Cell viabilities were calculated relative to positive control. Supernatants from cell lines adapted to cytotoxic drugs induced stronger HUVEC growth than supernatants from parental chemosensitive cells (Table 1). Moreover, the neuroblastoma cell lines UKF-NB-4 and Be(2)-C that were isolated as chemoresistant cell lines from patients materials induced stronger HUVEC growth than the chemosensitive parental cell lines UKF-NB-3, UKF-NB-2, or IMR-32. Subsequently, growth kinetics of HUVECs (determined by cell count) incubated with supernatants of UKF-NB-3, UKF-NB-3^r^VCR^10^, UKF-NB-3^r^CDDP^1000^, or UKF-NB-3^r^DOX^20 ^cells were compared confirming increased growth of HUVECs incubated with supernatants of chemoresistant cells (Figure 2A).

Next, the influence of neuroblastoma cell culture supernatants was examined on HUVEC survival. Confluent HUVEC monolayers were washed and incubated for 48 h with supernatants of UKF-NB-3, UKF-NB-3^r^VCR^10^, UKF-NB-3^r^CDDP^1000^, or UKF-NB-3^r^DOX^20 ^cells and HUVEC viability was determined. Results revealed increased HUVEC viability in cultures incubated with supernatants of chemoresistant cells (Table 1). Lack of growth factors or nutrients induces apoptosis in endothelial cells [26-28]. Therefore, we investigated caspase 3/7 activation as indicator of apoptosis in confluent HUVEC monolayers incubated for 48 h with supernatants of UKF-NB-3, UKF-NB-3^r^VCR^10^, UKF-NB-3^r^CDDP^1000 ^cells or UKF-NB-3^r^DOX^20 ^cells. Results indicated decreased caspase activation in HUVECs incubated with supernatants from chemoresistant cells (Figure 2B).

### Influence of neuroblastoma cell line supernatants on endothelial cell tube formation

**Figure 3:**
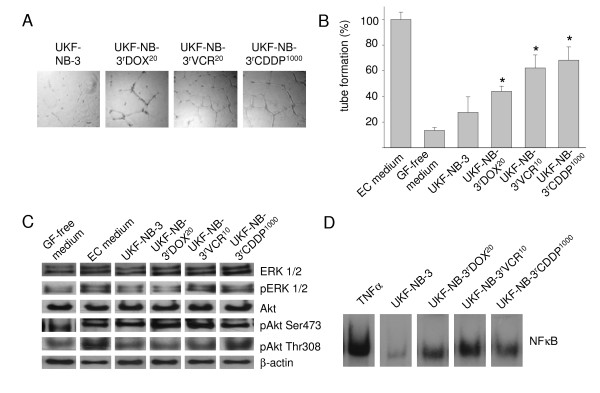
**Influence of neuroblastoma cell culture supernatants on tube formation and activation of pro-angiogenic signalling pathways in endothelial cells**. A) Representative photographs showing tube formation of human umbilical vein endothelial cells (HUVECs) suspended in 1:1 mixtures of IMDM + 10 FCS and supernatants of parental chemosensitive UKF-NB-3 cells or sublines adapted to vincristine (UKF-NB-3^r^VCR^10^), cisplatin (UKF-NB-3^r^CDDP^1000^), or doxorubicin (UKF-NB-3^r^DOX^20^) 16 h after seeding on extracellular matrix. B) Quantification of tube formation by counting of branching points of HUVEC incubated with 1:1 mixtures of IMDM and supernatants of UKF-NB-3, UKF-NB-3^r^VCR^10^, UKF-NB-3^r^CDDP^1000^, or UKF-NB-3^r^DOX^20 ^cells + 10% FCS in comparison to endothelial cell medium (EC medium, IMDM + 15% FCS + 5% pooled human serum + bFGF 2.5 ng/ml) and growth factor-free medium (GF-free medium, IMDM + 10% FCS). P < 0.05 relative to endothelial cells incubated with UKF-NB-3 supernatants. C) Representative Western blot showing expression of ERK 1/2, phosphorylated ERK 1/2 (pERK 1/2), Akt, Akt phosphorylated at Ser473 (pAkt Ser473), or Akt phosphorylated at Thr308 (pAkt Thr308) in endothelial cells incubated with 1:1 mixtures of IMDM and supernatants of UKF-NB-3, UKF-NB-3^r^VCR^10^, UKF-NB-3^r^CDDP^1000^, or UKF-NB-3^r^DOX^20 ^cells + 10% FCS in comparison to EC medium and GF-free medium for 24 h. β-actin was used as loading control. D) Representative EMSA showing nuclear factor κB (NFκB) activation in endothelial cells incubated with 1:1 mixtures of IMDM and supernatants of UKF-NB-3, UKF-NB-3^r^VCR^10^, UKF-NB-3^r^CDDP^1000^, or UKF-NB-3^r^DOX^20 ^cells + 10% FCS for 24 h. Tumour necrosis factor α (TNFα) was used as positive control.

HUVECs were suspended with supernatants of neuroblastoma cell lines and seeded on extracellular matrix (Matrigel). After 16 h, tube formation was determined. Results indicated increased tube formation in HUVECs suspended in supernatants of UKF-NB-3^r^VCR^10^, UKF-NB-3^r^CDDP^1000^, or UKF-NB-3^r^DOX^20 ^cells in comparison to HUVECs suspended in supernatants of the parenal chemosensitive UKF-NB-3 cell line (Figure 3A, B). Similar results were detected in the parental cell lines IMR-32 and UKF-NB-2 in comparison to their chemoresistant sub-lines [see Additional file 7]. Using different ratios of supernatants from the cell lines UKF-NB-3^r^VCR^10 ^or UKF-NB-3^r^CDDP^1000 ^and IMDM indicated that the supernatants induce tube formation in a concentration-dependent manner [see Additional file 7].

### Influence of neuroblastoma cell line supernatants on activation of pro-angiogenic signalling events in endothelial cells

The phosphoinositide-3-kinase (PI3K) - Akt (also known as protein kinase B, PKB) signalling pathway, "classical" mitogen-activated protein kinase (MAPK) signalling via Ras-Raf-MEK-ERK, and activation of nuclear factor κB (NFκB) are involved in angiogenesis signalling in endothelial cells [29-31]. The influence of supernatants of chemoresistant cells on Akt phosphorylation or ERK 1/2 phosphorylation in HUVECs is shown in Figure 3C. Densitometric analysis of Western blot data is given in Additional file 8. Akt may be activated through phosphorylation at Ser473 and/or at Thr308. The supernatants of UKF-NB-3^r^VCR^10 ^or UKF-NB-3^r^CDDP^1000 ^cells induced enhanced Akt phosphorylation at Thr308 and ERK 1/2 phosphorylation in comparison to UKF-NB-3 supernatants. All supernatants of chemoresistant cells caused enhanced NFκB activation relative to supernatants of chemosensitive UKF-NB-3 cells (Figure 3D).

### Chemoresistant cancer cells induce increased vessel formation in animal models

**Figure 4:**
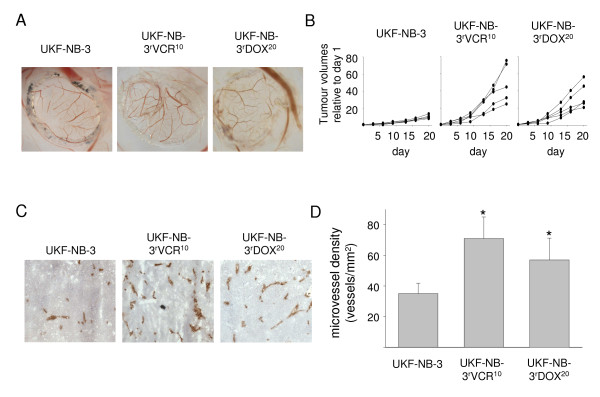
**Vessel formation induced by neuroblastoma cells in vivo**. A) Representative photographs of vessels induced by neuroblastoma cell lines in the chick chorioallantoic membrane. B) Growth curves of UKF-NB-3, UKF-NB-3^r^VCR^10^, or UKF-NB-3^r^DOX^20 ^tumours in nu/nu mice; UKF-NB-3^r^VCR^10 ^and UKF-NB-3^r^DOX^20 ^cells formed statistically significant larger tumours than UKF-NB-3 cells C) Representative photographs of angiogenesis in UKF-NB-3 or UKF-NB-3^r^VCR^10 ^xenograft tumours in nu/nu mice indicated by red (anti-CD31 antibody-stained) vessels. D) Microvessel density in UKF-NB-3 or UKF-NB-3^r^VCR^10 ^xenograft tumours in nu/nu mice. * P < 0.05 relative to UKF-NB-3 tumours.

Vessel formation was first investigated in vivo in the CAM of fertilised eggs. 10^6 ^tumour cells were seeded onto the CAM per egg (eight eggs per cell line) at day 10. Vessel formation was scored by two independent observers at day 14. Results indicated higher vessel formation in chemoresistant (UKF-NB-3^r^VCR^10^, UKF-NB-3^r^DOX^20^) cells than in chemosensitive (UKF-NB-3) cells (Figure 4A).

Vessel formation was further investigated in xenografts formed of UKF-NB-3, UKF-NB-3^r^VCR^10^, or UKF-NB-3^r^DOX^20 ^cells in female NMRI:nu/nu mice. Tumour take in mice injected with UKF-NB-3^r^VCR^10 ^cells was 100%, in mice injected with UKF-NB-3^r^DOX^20 ^cells it was 90% while only 10% of UKF-NB-3 cell-injected mice formed tumours. UKF-NB-3^r^VCR^10 ^cells and UKF-NB-3^r^DOX^20 ^cells also formed considerably bigger and stronger vascularised xenograft tumours than UKF-NB-3 cells (Figure 4B-D).

### Increased pro-angiogenic activity of chemoresistant neuroblastoma cells is mediated by individual molecular mechanisms

VEGF is a pro-angiogenic factor that has frequently been associated with neuroblastoma angiogenesis [32,33]. However, increased VEGF levels were not consistently found in supernatants of chemoresistant cells [see Additional file 9]. Acute cisplatin treament has been described to induce tumour progression through VEGF expression in paediatric tumour cells including the neuroblastoma cell line SK-N-BE2 [34]. In cisplatin-resistant neuroblastoma cells, VEGF expression has not been investigated, yet. Increased VEGF levels were detected in UKF-NB-3^r^CDDP^1000 ^cells versus UKF-NB-3 cells and in IMR-32^r^CDDP^1000 ^cells versus IMR-32 cells but not in UKF-NB-2^r^CDDP^10 ^cells versus UKF-NB-2 cells [see Additional file 9]. Moreover, the pro-angiogenic factors interleukin-8 (IL-8), angiogenin, basic fibroblast growth factor, or tumour necrosis factor α (TNF-α) were not generally found to be increased in supernatants of chemoresistant cells [see Additional file 9]. Two angiogenesis-associated genes (ARHGAP8, FGFR2) were found commonly up-regulated in UKF-NB-3^r^CDDP^1000^, UKF-NB-3^r^VCR^10^, or UKF-NB-3^r^DOX^20 ^cells versus UKF-NB-3 cells [see Additional files 2, 3, 6]. However, these genes were not consistently found up-regulated in chemoresistant neuroblastoma cells (data not shown).

Expression of a number of further pro- and anti-angiogenic factors has been suggested to be relevant for neuroblastoma angiogenesis including platelet-derived growth factor α (PDGFα), matrix metalloproteinase 2 (MMP-2), MMP-9, erythropoietin (EPO), EPO receptor, activin A, interleukin-6 (IL-6), leukemia inhibitory factor (LIF), tissue inhibitor of metalloproteinase 2 (TIMP2), pigment epithelial-derived growth factor (PEDGF), secreted protein acidic and rich in cysteine (SPARC), thrombospondin-1, and thrombospondin-2 [33]. However, analysis of gene microarray data from neuroblastoma cell lines did not reveal specific expression of these or other angiogenesis-related genes that would suggest a single common molecular event underlying increased neuroblastoma tumour angiogenesis in all chemoresistant cells (data not shown).

N-myc amplification has also been reported to result in increased neuroblastoma tumour angiogenesis through different mechanisms [33,35-37]. However, UKF-NB-3^r^DOX^20 ^cells showed enhanced pro-angiogenic potential compared to UKF-NB-3 cells although both cell lines do neither differ in N-myc amplification nor in N-myc expression [8,18]. This indicates that the N-myc status may not generally be critical for increased pro-angiogenic potential of chemoresistant cells. Furthermore, the loss of functional p53 during tumourigenesis has been correlated to a more pro-angiogenic tumour phenotype [38]. However, in our experiments pro-angiogenic activity was enhanced in both p53-mutated and p53-wild-type chemoresistant neuroblastoma cells (Table 1). Taken together, the more pro-angiogenic phenotype observed in chemoresistant neuroblastoma cells appears to result from different individual shifts in the expression of angiogenesis-associated genes.

### Doxorubicin inhibits tumour angiogenesis and growth of doxorubicin-resistant neuroblastoma xenografts

Data had indicated individual changes in the expression of angiogenesis-related genes to be responsible for the proangiogenic phenotype of chemoresistant neuroblastoma cells (see above). To investigate if the increased pro-angiogenic activity of chemoresistant neuroblastoma cells may be relevant for enhanced growth of chemoresistant neuroblastoma xenografts, doxorubicin-resistant UKF-NB-3^r^DOX^20 ^neuroblastoma cells were treated with doxorubicin that is known to interfere with angiogenesis by direct influence on endothelial cells [39,40].

**Figure 5:**
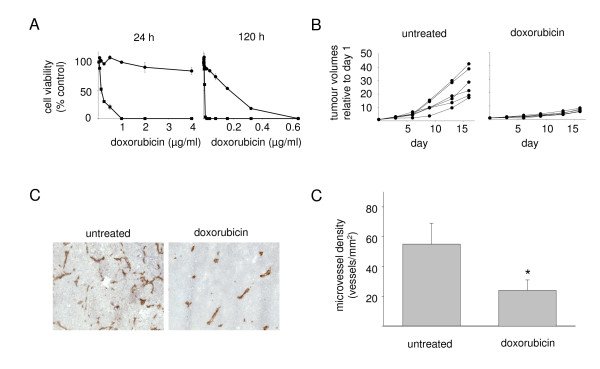
**Influence of doxorubicin on viability of neuroblastoma cells, growth of doxorubicin-resistant neuroblastoma xenografts, and vessel formation in doxorubicin-resistant neuroblastoma xenografts**. A) Dose-response curves of parental chemosensitive neuroblastoma cell cultures (UKF-NB-3; black square) or the doxorubicin-resistant sub-line (UKF-NB-3^r^DOX^20^; black dot) after 24 h or 120 h incubation periods. B) Tumour volumes in mice injected with 10^7 ^UKF-NB-3^r^DOX^20 ^cells; doxorubicin treatment (8 mg/kg i.v.) was performed at day 1, i.e. when tumours became palpable. C) Representative photographs of angiogenesis in untreated or doxorubicin-treated UKF-NB-3^r^DOX^20 ^xenograft tumours by red (anti-CD31 antibody-stained) vessels at day 16. D) Microvessel density in untreated or doxorubicin-treated UKF-NB-3^r^DOX^20 ^xenografts at day 16. * P < 0.05 relative to UKF-NB-3 tumours.

Administration of a single dose of doxorubicin 10 mg/kg i.v. into mice results in maximal doxorubicin plasma levels in the range of 500 - 600 ng/ml that decline to doxorubicin plasma levels of 20 - 30 ng/ml 24 h after injection [40-42]. One time application of doxorubicin 8 mg/kg i.v. resulted in intratumoural doxorubicin concentrations of about 10 - 20 ng/ml in a melanoma xenograft model [43]. The doxorubicin IC_50 _values of UKF-NB-3^r^DOX^20 ^cells are > 4000 ng/ml after a 24 h incubation period and 180.50 ± 22.13 ng/ml after 120 h incubation period. Dose response curves for doxorubicin treatment of UKF-NB-3^r^DOX^20 ^cells are shown in comparison to parental chemosensitive UKF-NB-3 cells in Figure 5A. Consequently, treatment of UKF-NB-3^r^DOX^20 ^xenograft carrying mice with doxorubicin 8 mg/kg i.v. should not directly affect UKF-NB-3^r^DOX^20 ^cell viability and tumour growth. Therefore, mice received doxorubicin 8 mg/kg i.v. when tumours became palpable and tumour volumes were observed for 16 days. Then mice were sacrificed and xenograft tumours were examined for vessel density. Doxorubicin strongly reduced UKF-NB-3^r^DOX^20 ^xenograft growth as well vessel density in the tumours (Figure 5B-D). TUNEL staining indicated an increase in the number of apoptotic cells in doxorubicin-treated (231 ± 61 cells per microscopic field at 200× magnification) vs. non-treated UKF-NB-3^r^DOX^20 ^(112 ± 22 cells per microscopic field at 200× magnification) xenografts. The fraction of ki67-expressing proliferating cells was higher in non-treated tumours (163 ± 26 cells per microscopic field at 200× magnification) than in doxorubicin-treated tumours (101 ± 14% cells per microscopic field at 200× magnification) indicating decreased proliferation.

## Discussion

Here, we used a bioinformatics-based approach based on transcriptomics data to identify signalling pathways associated with increased malignant behaviour of chemoresistant neuroblastoma cells. Angiogenesis signalling belonged to the top 5 pathways most strongly differentially regulated between chemosensitive and chemoresistant neuroblastoma cells. Systematic evaluation of a panel of neuroblastoma cell lines in cell culture and animal models showed consitently increased pro-angiogenic acivity exerted by chemoresistant cells. These findings are in accordance with previous reports showing that human melanoma and breast cancer cells selected for resistance to chemotherapeutic agents produced higher levels of multiple angiogenic factors [44,45]. Moreover, an increased microvessel density (MVD) was detected in chemotherapy resistant xenograft tumours [44,45].

Selection of cancer stem cells has been suggested to play a role in the enhanced pro-angiogenic activity seen in chemoresistant cancer cells. In lung cancer cells, treatment with cisplatin, doxorubicin, or etoposide resulted in the selection of cancer stem cells as indicated by cell biology and analysis of expression of stemness genes [46]. These chemotherapy-selected cancer stem cells were responsible for the observed increased pro-angiogenic properties of lung cancer cells. In the absence of cytotoxic drugs, lung cancer cell lines returned to their initial phenotype and re-acquired drug sensitivity [46]. In contrast, UKF-NB-3^r^VCR^10 ^and UKF-NB-3^r^CDDP^1000 ^cells remained chemoresistant and did not loose their pro-angiogenic phenotype even when they were cultivated for up to six months in the absence of drugs (data not shown). This suggests that chemoresistance and pro-angiogenic activity in these cell lines are not consequence of a simple chemotherapy-induced selection of cancer stem cells that are already present in the parental UKF-NB-3 cell line. Moreover, acute cisplatin treatment increased VEGF expression together with expression of the stemness genes Nanog, Bmi-1, and Oct 4 in osteosarcoma (HOS), rhabdomyosarcoma (RH-4) and neuroblastoma (SK-N-BE2) cell lines [46]. However, none of these stemness genes was found up-regulated in UKF-NB-3^r^VCR^10 ^or UKF-NB-3^r^CDDP^1000 ^cells relative to UKF-NB-3 cells [see Additional file 10].

The finding that cell culture supernatants from chemoresistant cells exerted stronger pro-angiogenic effects than those from chemosensitive cells suggests that soluble factors contribute to the enhanced pro-angiogenic activity exerted by chemoresistant neuroblastoma cells. Statistical analysis of the expression of angiogenesis-related genes indicated clear differences between chemosensitive UKF-NB-3 cells and the chemoresistant sub-lines UKF-NB-3^r^VCR^10^, UKF-NB-3^r^CDDP^1000^, or UKF-NB-3^r^DOX^20 ^(Figure 1) [see Additional files 1, 4, 5]. Obviously, chemoresistance development resulted in a global change of expression of angiogenesis-associated genes towards a more pro-angiogenic phenotype. The resistance-related changes in expression patterns appear to differ between individual chemoresistant neuroblastoma cell lines. This suggests that the enhanced pro-angiogenic phenotype observed in all chemoresistant neuroblastoma cell lines in comparison to the chemosensitive cell lines is caused by different (individual) changes in the expression patterns of angiogenesis-associated genes. Notably, hierarchical clustering of expression of angiogenesis-associated genes also clearly discriminated UKF-NB-2 cells from UKF-NB-2^r^VCR^10 ^and UKF-NB-2^r^CDDP^1000 ^cells [see Additional file 11], as well as IMR-32 cells from IMR-32^r^VCR^10 ^cells [see Additional file 12].

The view that individual chemoresistant neuroblastoma cell lines exert pro-angiogenic effects by individual mechanisms is supported by the results derived from the examination of pro-angiogenic signalling in endothelial cells incubated with supernatants from different neuroblastoma cell lines. Supernatants of chemoresistant UKF-NB-3^r^DOX^20^, UKF-NB-3^r^VCR^10^, and UKF-NB-3^r^CDDP^1000 ^cells enhanced NFκB activation compared to supernatants of chemosensitive UKF-NB-3 cells However, only supernatants of UKF-NB-3^r^VCR^10 ^and UKF-NB-3^r^CDDP^1000 ^cells but not UKF-NB-3^r^DOX^20 ^cells elevated Akt and ERK 1/2 phosphorylation in endothelial cells. Based on these differences in the activation of pro-angiogenic signalling events in endothelial cells, it appears plausible that endothelial cell activation might be caused by different chemoresistant neuroblastoma cell lines by different molecular mechanisms resulting in up- or down-regulation of varying pro- or anti-angiogenic factors.

Possibly, there is an overlap between gene products involved in angiogenesis and gene products relevant in chemoresistance. Indeed, among between the aniogenesis-associated genes that were differentially expressed there are those that are also considered to contribute to chemoresistance. Three arbitrarily chosen examples are BIRC5, MAPK3, and AKT1. BIRC5 (increased expression in UKF-NB-3^r^VCR^10 ^vs. UKF-NB-3, [see Additional file 2], and in UKF-NB-3^r^DOX^20 ^vers. UKF-NB-3, [see Additional file 6]) encodes for a protein that is also named survivin and plays a prominent role in apoptosis inhibition and cancer cell chemoresistance [47]. Moreover, BIRC5 expression in cancer cells has been linked to tumour angiogenesis [48] and inhibition of BIRC5 expression in tumour cells decreased tumour angiogenesis [49,50]. MAPK3 (increased expression in UKF-NB-3^r^VCR^10 ^vs. UKF-NB-3, [see Additional file 2]) encodes for a protein that is also called extracellular signal-regulated kinase 1 (ERK1) and is a constituent of the "classical" MAP kinase pathway Ras/Raf/MEK/ERK. ERK1 phosphorylation protects cancer cells from different entities against chemotherapy-induced apoptosis [51-53]. Moreover, MAPK3 activation/phosphorylation induces production of pro-angiogenic factors in renal carcinoma cells [54]. AKT1 (increased expression in UKF-NB-3^r^DOX^20 ^vers. UKF-NB-3, [see additional file 6]) encodes for a protein also called protein kinase B (PKB) that is a central mediator of survival signals transduced by the phosphatidylinositol 3-kinase and is involved in chemoresistance [55-57] as well as in cancer cell expression of pro-angiogenic factors [58-60]. Remarkably, an angiogenesis-associated gene expression signature had been described before to predict the sensitivity of cancer cells to artemisinins, an anti-cancer active group of anti-malaria drugs [61].

The complexicity of pro-angiogenic mechanisms observed in chemoresistant neuroblastoma cells is in accordance with other reports demonstrating that pro-angiogenic activity of cancer cells is commonly caused by complex changes in angiogenesis signalling and that inhibition of one pro-angiogenic event may not be enough to interfere with tumour vessel formation [62]. N-myc-amplified neuroblastoma cells that exert pro-angiogenic activity mainly through VEGF have very recently been shown to rapidly develop alternative pro-angiogenic mechanisms when VEGF signalling is inhibited [63]. In addition, up-regulation of multiple pro-angiogenic factors enabled carcinoma cells to escape from angiogenesis inhibition by the three endogenous anti-angiogenic molecules thrombospondin-1, endostatin, and tumstatin [64]. Notably, combination therapy of metastatic breast cancer with paclitaxel and the anti-VEGF-A antibody bevacizumab resulted in prolonged progression-free survival but did not influence overall survival relative to paclitaxel in a phase III trial [65]. In the light of the findings presented here, one may speculate that anti-angiogenic therapy may prolong progression-free survival but that resistance development (to chemotherapy and/or anti-angiogenic therapy) may result in a more aggressive cancer cell phenotype, which might be the reason for the decreased time period observed between tumour re-onset and patients' deaths.

High tumour angiogenesis and high-level expression of pro-angiogenic factors at diagnosis have previously been suggested to be correlated with advanced disease stages in neuroblastoma [32,66,67]. However, the prognostic value of angiogenesis in neuroblastoma at diagnosis is still a matter of debate [68,69]. Notably, analysis of two different data sets reporting on gene expression profiles in tumours from poor outcome or bad outcome N-myc amplified [70] or non-N-myc amplified [71] neuroblastoma patients indicated statistically significant differences in angiogenesis signalling between these groups [see Additional files 13, 14]. To investigate if the increased pro-angiogenic phenotype observed in chemoresistant cells may contribute to tumour progression, xenografts grown from doxorubicin-resistant (UKF-NB-3^r^DOX^20^) cells were treated with doxorubicin, an anti-cancer drug that exerts anti-angiogenic activity by direct effect on endothelial cells [39,40]. Tumour vessel formation and growth were strongly reduced by doxorubicin in doxorubicin-resistant xenografts. Although it cannot be concluded without a doubt that the entire effect on xenograft growth can be attributed to inhibition of angiogenesis, microvessel density was statistically reduced supporting the view that inhibition of angiogenesis has definitely contributed. Therefore, these data suggest that increased pro-angiogenic activity of doxorubicin-resistant cells contributes to their more malignant phenotype and that anti-angiogenic strategies that target endothelial cells might represent a therapeutic option for neuroblastoma treatment.

## Conclusion

Bioinformatics pathway analysis indicated differences in the expression of angiogenesis-associated genes between chemosensitive and chemoresistant neuroblastoma cell lines. Cell culture and animal data showed that acquired resistance to different anti-cancer drugs resulted in increased pro-angiogenic activity of neurobastoma cells. The changes in angiogenesis signalling observed in chemoresistant neuroblastoma cells are very complex and differ between individual cell lines. Therefore, individual molecular mechanisms appear to be responsible for the enhanced pro-angiogenic activity that was consistently observed in all investigated chemoresistant neuroblastoma cell lines relative to chemosensitive cells. Doxorubicin treatment of doxorubicin-resistant neuroblastoma xenografts resulted in decreased vessel formation and tumour growth suggesting that the more pro-angiogenic phenotype of chemoresistant cells may contribute to increased malignancy of chemoresistant neuroblastoma cells and that endothelial cell targeting may represent a possibility for therapeutic intervention. The complex nature of the chemoresistance-associated changes responsible for the more pro-angiogenic phenotype strongly stresses the need for an improved understanding of biological processes like angiogenesis on a systems biology level.

## Competing interests

The authors declare that they have no competing interests.

## Authors' contributions

MM, JC, and JC jr. were involved in acquisition, conception, design, analysis, and interpretation of data and drafted the manuscript. DK, TS, and NH were involved in acquisition, analysis, and interpretation of data. SB was involved in design, analysis, and interpretation of data. TS was involved in acquisition, analysis, and interpretation of data. RB and BM were involved in analysis and interpretation of data and revised the manuscript critically for important intellectual content. HWD was involved in conception, design, analysis, and interpretation of data. All authors have given final approval of the version to be published.

## Supplementary Material

Additional file 1 **Bioinformatic pathway analysis in neuroblastoma cells**. Most strongly influenced signalling pathways between chemosensitive (UKF-NB-3) and chemoresistant (UKF-NB-3^r^VCR^10^, UKF-NB-3^r^CDDP^1000^) neuroblastoma cells. Click here for file

Additional file 2 **Expression analysis of angiogenesis-associated genes**. Angiogenesis-related genes that are differentially expressed between the parental chemosensitive UKF-NB-3 neuroblastoma cell line and the vincristine-resistant sub-line UKF-NB-3^r^VCR^10^. Click here for file

Additional file 3 **Expression analysis of angiogenesis-associated genes**. Angiogenesis-related genes that are differentially expressed between the parental chemosensitive UKF-NB-3 neuroblastoma cell line and the cisplatin-resistant sub-line UKF-NB-3^r^CDDP^1000^. Click here for file

Additional file 4 **Bioinformatic pathway analysis in neuroblastoma cells**. Most strongly influenced signalling pathways between the chemosensitive neuroblastoma cell line UKF-NB-3 and the doxorubicin-resistant sub-line UKF-NB-3^r^DOX^20^. Click here for file

Additional file 5 **Hierarchical cluster analysis of expresson of angiogenesis-associated genes**. Hierarchical cluster analysis and heatmap showing expression of angiogenesis-associated genes in UKF-NB-3 or UKF-NB-3^r^DOX^20 ^cells. Click here for file

Additional file 6 **Expression analysis of angiogenesis-associated genes**. Angiogenesis-related genes that are differentially expressed between the parental chemosensitive UKF-NB-3 neuroblastoma cell line and the doxorubicin resistant sub-line UKF-NB-3^r^DOX^20^. Click here for file

Additional file 7 **Endothelial cell tube formation induced by supernatants of neuroblastoma cells**. Quantification of endothelial cell tube formation induced by supernatants of neuroblastoma cells. Click here for file

Additional file 8 **Densitometric analysis of Western blot analyses**. Densitometric analysis of Western blot analyses investigating expression of ERK 1/2, phosphorylated ERK 1/2 (pERK 1/2), Akt, Akt phosphorylated at Ser473 (pAkt Ser473), or Akt phosphorylated at Thr308 (pAkt Thr308) in endothelial cells incubated supernatants of UKF-NB-3, UKF-NB-3^r^VCR^10^, UKF-NB-3^r^CDDP^1000^, or UKF-NB-3^r^DOX^20 ^cells. Click here for file

Additional file 9 **Pro-angiogenic factors in the supernatants of neuroblastoma cell lines**. Concentrations of selected pro-angiogenic factors in the supernatants of neuroblastoma cell lines. Click here for file

Additional file 10 **Expression of stemness genes**. Comparison of expression of selected stemness genes in chemoresistant or chemosensitive neuroblastoma cells. Click here for file

Additional file 11 **Hierarchical cluster analysis of expresson of angiogenesis-associated genes**. Hierarchical cluster analysis and heatmap showing expression of angiogenesis-associated genes in UKF-NB-2, UKF-NB-2^r^VCR^10^, or UKF-NB-2^r^CDDP^1000 ^cells. Click here for file

Additional file 12 **Hierarchical cluster analysis of expresson of angiogenesis-associated genes**. Hierarchical cluster analysis and heatmap showing expression of angiogenesis-associated genes in IMR-32 or IMR-32^r^VCR^10 ^cells. Click here for file

Additional file 13 **Bioinformatic pathway analysis in neuroblastoma patients' tumours**. Signalling pathways most strongly differentially regulated between N-myc amplified neuroblastoma tissues from patients with favourable outcome or poor outcome. Click here for file

Additional file 14 **Bioinformatic pathway analysis in neuroblastoma patients' tumours**. Signalling pathways most strongly differentially regulated between non-N-myc amplified neuroblastoma tissues from patients with favourable outcome or poor outcome. Click here for file
