# Supplementary material for: Chemoresistance acquisition induces a global shift of expression of aniogenesis-associated genes and increased pro-angogenic activity in neuroblastoma cells
Source: Mol Cancer. 2009 Sep 29;8:80. doi: 10.1186/1476-4598-8-80 (PMC2761864; doi:10.1186/1476-4598-8-80)
Supplement: Additional file 1 — Bioinformatic pathway analysis in neuroblastoma cells. Most strongly influenced signalling pathways between chemosensitive (UKF-NB-3) and chemoresistant (UKF-NB-3rVCR10, UKF-NB-3rCDDP1000) neuroblastoma cells. [file 1476-4598-8-80-S1.PDF]

Additional file 1. Most strongly influenced signalling pathways between chemosensitive (UKF-NB-3) and chemoresistant (UKF-NB-3^{r}VCR^{10}, UKF-NB-3^{r}CDDP^{1000}) neuroblastoma cells as indicated by PANTHER pathway analysis of gene microarray (AB1700) expression data.

**PANTHER signalling pathway**	**p-value**
1. TGF-β signalling pathway	1.48×10^{-5}
2. p38 MAPK pathway	2.59×10^{-5}
3. Ras pathway	1.79×10^{-4}
**4. Angiogenesis**	**1.87×10^{-4}**
5. EGF receptor signaling pathway	3.02×10^{-4}
