# Supplementary material for: Chemoresistance acquisition induces a global shift of expression of aniogenesis-associated genes and increased pro-angogenic activity in neuroblastoma cells
Source: Mol Cancer. 2009 Sep 29;8:80. doi: 10.1186/1476-4598-8-80 (PMC2761864; doi:10.1186/1476-4598-8-80)
Supplement: Additional file 2 — Expression analysis of angiogenesis-associated genes. Angiogenesis-related genes that are differentially expressed between the parental chemosensitive UKF-NB-3 neuroblastoma cell line and the vincristine-resistant sub-line UKF-NB-3rVCR10. [file 1476-4598-8-80-S2.PDF]

Additional file 2. Angiogenesis-related genes that are differentially expressed between the parental chemosensitive UKF-NB-3 neuroblastoma cell line and the vincristine-resistant sub-line UKF-NB-3^{r}VCR^{10} as indicated by PANTHER pathway analysis of gene microarray (AB1700) expression data.

**Comparison UKF-NB-3^{r}VCR^{10} versus UKF-NB-3**		
Gene ID	Encoded protein	Fold change<br>UKF-NB-3^{r}VCR^{10}/ UKF-NB-3<br>(multiple-testing corrected p-value based on three biological replicates)
ARHGAP8	Rho GTPase activating protein 8	6.16 (0.009)
JAK1	Janus kinase 1	5.27 (0.020)
NOTCH1	Notch1	4.14 (0.030)
FGFR2	FGF receptor 2	4.13 (0.019)
NRAS	neuroblastoma RAS viral (v-ras) oncogene homolog	3.77 (0.021)
WNT10B	wingless-type MMTV integration site family, member 10B;<br>WNT-10B protein	3.67 (0.035)
DVL1	dishevelled-1	2.91 (0.007)
RRAS	R-Ras	2.72 (0.036)
CASP9	caspase 9	2.42 (0.026)
BIRC5	survivin	2.27 (0.008)<br>1.89 (0.010)
PDGFRB	platelet-derived growth factor receptor beta; PDGFRβ	2.20 (0.017)
RHOC	RhoC	2.13 (0.047)
STAT3	Signal transducer and activator of transcription 3	2.11 (0.028)<br>1.61 (0.043)

SHC1	SHC (Src homology 2 domain containing) transforming protein 1	2.07 (0.016)
MAPK12	ERK3<br>ERK5<br>ERK6<br>p38γ<br>SAPK3	2.04 (0.014)
FGFR3	Fibroblast growth factor receptor 3<br>CD133	1.97 (0.021)
MAPK3	Mitogen-activated protein kinase 3;<br>MAPK1;<br>Extracellular signal-regulated kinase 1 (ERK1);<br>p44	1.75 (0.047)
PIK4CB	phosphatidylinositol 4-kinase β;<br>phosphatidylinositol 4-kinase, catalytic, β;<br>PI4K-β	1.65 (0.044)
MAPK14	mitogen-activated protein kinase 14;<br>p38α	1.63 (0.024)
AXIN1	axin 1;<br>axis inhibitor 1;<br>axis inhibitor protein 1	1.52 (0.026)
ABR	Active breakpoint cluster region-related protein;<br>active BCR-related gene;<br>MDB	1.52 (0.015)
STAT1	signal transducer and activator of transcription 1;<br>STAT91;<br>ISGF-3	1.51 (0.021)
HIF1A	Hypoxia-inducible factor 1 alpha;<br>ARNT interacting protein	1.47 (0.016)
PXN	paxillin	1.45 (0.022)
GRB2	growth factor receptor-bound protein 2;<br>EGFRBP-GRB2; growth factor receptor-bound protein 3	1.43 (0.011)

BCR	breakpoint cluster region	1.36 (0.019)
RHOA	ras homolog gene family, member A;<br>small GTP binding protein RhoA;<br>RhoA<br>Rho12	1.25 (0.030)
null	unknown gene	0.69 (0.014)
FZD3	frizzled 3;<br>frizzled homolog 3 (Drosophila)	0.68 (0.013)
CTNNB1	β-catenin;<br>catenin β -1;<br>catenin (cadherin-associated protein), beta 1 (88kD)	0.55 (0.021)
PDGFA	platelet-derived growth factor α;<br>PDGF A-chain;<br>PDGF1	0.53 (0.019)
WNT5B	wingless-type MMTV integration site family, member 5B;<br>WNT-5B protein	0.32 (0.029)
YES1	v-yes-1 Yamaguchi sarcoma viral oncogene homolog 1;<br>c-Yes;<br>Yamaguchi sarcoma oncogene;<br>Proto-oncogene tyrosine-protein kinase Yes	0.31 (0.007)<br>0.28 (0.009)
PLA2G4E	Phospholipase A2 group IVE;<br>Cytosolic phospholipase A2 epsilon;<br>cPLA2ε	0.29 (0.026)
FZD2	frizzled 2	0.26 (0.014)
FRZB	Frizzled- related protein 1;<br>FRZB1;<br>Frezzled;<br>frizzled-related protein;<br>FRP;<br>FRP-3;<br>Fritz;<br>Secreted frizzled related protein 1;<br>SFRP1	0.24 (0.010)
