# Supplementary material for: Chemoresistance acquisition induces a global shift of expression of aniogenesis-associated genes and increased pro-angogenic activity in neuroblastoma cells
Source: Mol Cancer. 2009 Sep 29;8:80. doi: 10.1186/1476-4598-8-80 (PMC2761864; doi:10.1186/1476-4598-8-80)
Supplement: Additional file 3 — Expression analysis of angiogenesis-associated genes. Angiogenesis-related genes that are differentially expressed between the parental chemosensitive UKF-NB-3 neuroblastoma cell line and the cisplatin-resistant sub-line UKF-NB-3rCDDP1000. [file 1476-4598-8-80-S3.PDF]

Additional file 3. Angiogenesis-related genes that are differentially expressed between the parental chemosensitive UKF-NB-3 neuroblastoma cell line and the cisplatin-resistant sub-line UKF-NB-3^{r}CDDP^{1000} as indicated by PANTHER pathway analysis of gene microarray (AB1700) expression data.

**Comparison UKF-NB-3^{r}CDDP^{1000} versus UKF-NB-3**		
Gene ID	Encoded protein	Fold change UKF-NB-3^{r}CDDP^{1000}/UKF-NB-3 (multiple-testing corrected p-value based on three biological replicates)
ARHGAP8	Rho GTPase activating protein 8	24.21 (0.041)
FGFR2	FGF receptor 2	11.66 (0.047)
WNT2B	wingless-type MMTV integration site family, member 2B;<br>Wnt-2b;<br>wingless-type MMTV integration site family, member 13;<br>WNT13;<br>Wnt-13	7.36 (0.043)
DLL1	delta-like 1 (Drosophila);	6.70 (0.049)
WNT10B	wingless-type MMTV integration site family, member 10B;<br>WNT-10B protein	5.37 (0.049)
RRAS	R-Ras	5.26 (0.040)
PDGFRB	platelet-derived growth factor receptor beta; PDGFRβ	5.03 (0.043)
DLL4		1.59 (0.037)
PAK7	Serine/threonine-protein kinase PAK 7;<br>p21(CDKN1A)-activated kinase 7;<br>p21-activated kinase 7;<br>PAK-7	0.10 (0.049)

	protein kinase PAK5;<br>PAK5;<br>PAK-5	
GRB10	growth factor receptor-bound protein 10;<br>Grb-10<br>GRB10 adaptor protein;<br>Insulin receptor-binding protein GRB-IR;<br>GRB-IR;<br>GRBIR;<br>maternally expressed gene 1;<br>MEG1	0.03 (0.046)
