# Supplementary material for: Chemoresistance acquisition induces a global shift of expression of aniogenesis-associated genes and increased pro-angogenic activity in neuroblastoma cells
Source: Mol Cancer. 2009 Sep 29;8:80. doi: 10.1186/1476-4598-8-80 (PMC2761864; doi:10.1186/1476-4598-8-80)
Supplement: Additional file 4 — Bioinformatic pathway analysis in neuroblastoma cells. Most strongly influenced signalling pathways between the chemosensitive neuroblastoma cell line UKF-NB-3 and the doxorubicin-resistant sub-line UKF-NB-3rDOX20. [file 1476-4598-8-80-S4.PDF]

Additional file 4. Most strongly influenced signalling pathways between the chemosensitive neuroblastoma cell line UKF-NB-3 and the doxorubicin-resistant sub-line UKF-NB-3^{r}DOX^{20} as indicated by PANTHER pathway analysis of gene microarray (HGU133 Plus 2.0) expression data.

**PANTHER signalling pathway**	**p-value**
1. Wnt signaling pathway	3.04×10^{-7}
2. Cadherin signaling pathway	9.39×10^{-7}
3. Ras pathway	2.38×10^{-6}
**4. Angiogenesis**	$\mathbf{5.54 \times 10^{-6}}$
5. B cell activation	2.20×10^{-3}
