# Supplementary material for: Chemoresistance acquisition induces a global shift of expression of aniogenesis-associated genes and increased pro-angogenic activity in neuroblastoma cells
Source: Mol Cancer. 2009 Sep 29;8:80. doi: 10.1186/1476-4598-8-80 (PMC2761864; doi:10.1186/1476-4598-8-80)
Supplement: Additional file 5 — Hierarchical cluster analysis of expresson of angiogenesis-associated genes. Hierarchical cluster analysis and heatmap showing expression of angiogenesis-associated genes in UKF-NB-3 or UKF-NB-3rDOX20 cells. [file 1476-4598-8-80-S5.PDF]

# Additional file 5

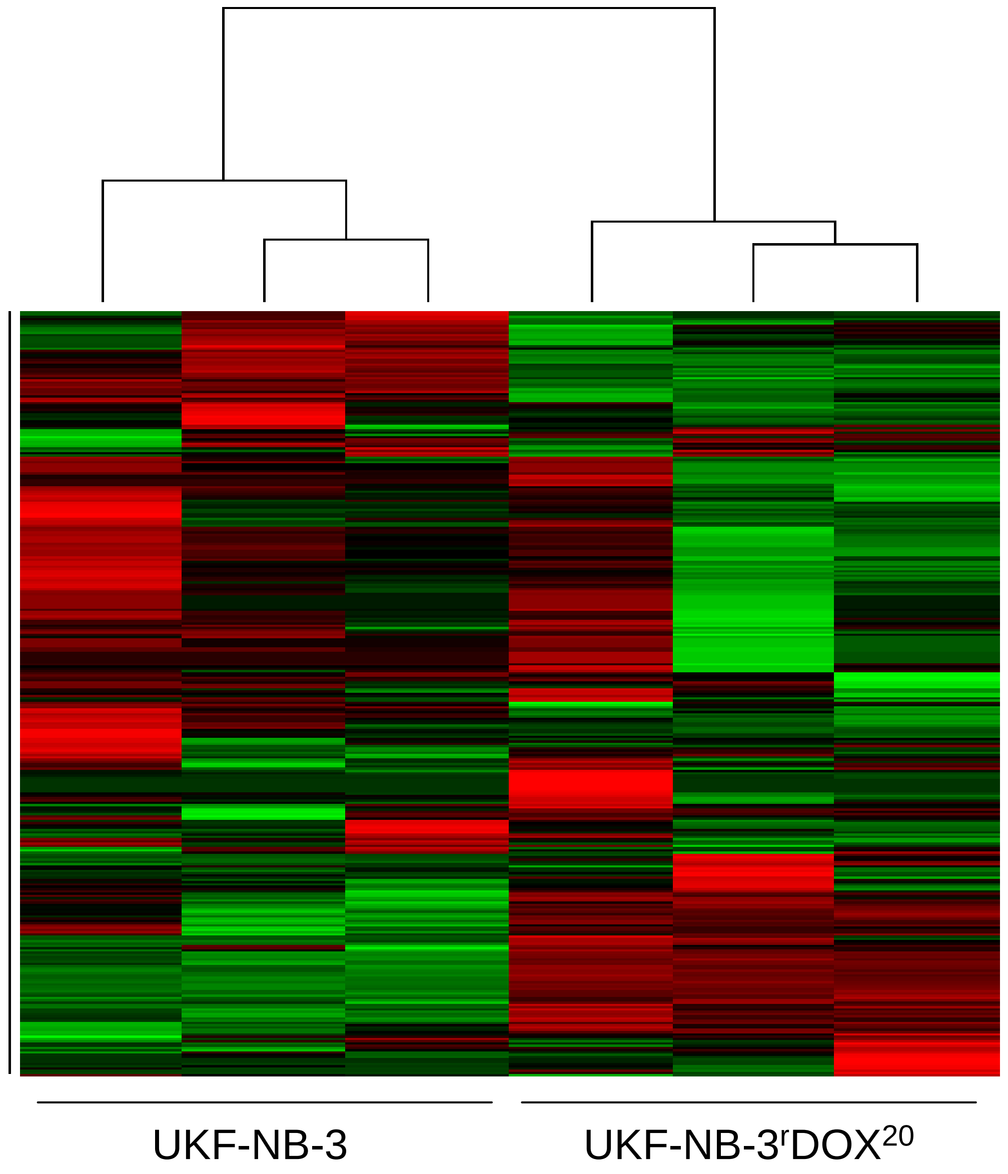


**Additional file 5.** Hierarchical cluster analysis and heatmap showing expression of angiogenesis-associated genes (taken from PANTHER pathway) in UKF-NB-3 or UKF-NB-3$^r$DOX$^{20}$ cells.
