# Supplementary material for: Chemoresistance acquisition induces a global shift of expression of aniogenesis-associated genes and increased pro-angogenic activity in neuroblastoma cells
Source: Mol Cancer. 2009 Sep 29;8:80. doi: 10.1186/1476-4598-8-80 (PMC2761864; doi:10.1186/1476-4598-8-80)
Supplement: Additional file 6 — Expression analysis of angiogenesis-associated genes. Angiogenesis-related genes that are differentially expressed between the parental chemosensitive UKF-NB-3 neuroblastoma cell line and the doxorubicin resistant sub-line UKF-NB-3rDOX20. [file 1476-4598-8-80-S6.PDF]

Additional file 6. Angiogenesis-related genes that are differentially expressed between the parental chemosensitive UKF-NB-3 neuroblastoma cell line and the doxorubicin resistant sub-line UKF-NB-3^{r}DOX^{20} as indicated by PANTHER pathway analysis of gene microarray (HGU133 Plus 2.0) expression data.

**Comparison UKF-NB-3 against UKF-NB-3^{r}DOX^{20}**		
Gene ID	Encoded protein	Fold change UKF-NB-3^{r}DOX^{20}/UKF-NB-3 (multiple-testing corrected p-value based on three biological replicates)
DLK1	delta-like 1 homolog (Drosophila)	282,14 (2.85 x 10^{-9})
TCF7L2	transcription factor 7-like 2 (T-cell specific, HMG-box)	5,72 (4.30 x 10^{-4})<br>3,11 (1.57 x 10^{-4})<br>2,47 (7.40 x 10^{-3})<br>1,74 (1.80 x 10^{-4})<br>1,74 (0.03)
DLL1	delta-like 1 (Drosophila)	4,97 (1.79 x 10^{-5})
SHC1	SHC (Src homology 2 domain containing) transforming protein 1	3,90 (3.55 x 10^{-6})<br>2,11 (1.90 x 10^{-5})
FGFR3	fibroblast growth factor receptor 3 (achondroplasia, thanatophoric dwarfism)	3,75 (6.57 x 10^{-6})<br>1.10 (0.03)
NOTCH3	Notch homolog 3 (Drosophila)	2,99 (4.01 x 10^{-3})
SFRP1	secreted frizzled-related protein 1	2,89 (2.40 x 10^{-4})<br>2,88 (1.83 x 10^{-4})<br>2,62 (1.74 x 10^{-5})
MAPK9	mitogen-activated protein kinase 9	2,69 (5.76 x 10^{-5})<br>1,83 (1.60 x 10^{-4})
DLL3	delta-like 3 (Drosophila)	2,42 (1.64 x 10^{-3})<br>2,41 (3.66 x 10^{-3})
AKT2	v-akt murine thymoma viral oncogene homolog 2	2,33 (1.74 x 10^{-3})<br>2,30 (1.25 x 10^{-4})<br>1,93 (2.89 x 10^{-3})

		1.33 (0.01)<br>1.17 (0.01)
FGFR2	fibroblast growth factor receptor 2 (bacteria-expressed kinase, keratinocyte growth factor receptor, craniofacial dysostosis 1, Crouzon syndrome, Pfeiffer syndrome, Jackson-Weiss syndrome)	2,20 (5.30 x 10^{-4})<br>1,89 (2.42 x 10^{-4})
GRB2	growth factor receptor-bound protein 2	2,20 (3.69 x 10^{-4})<br>1,82 (2.28 x 10^{-4})
FGFR1	fibroblast growth factor receptor 1 (fms-related tyrosine kinase 2, Pfeiffer syndrome)	2,08 (4.36 x 10^{-3})<br>1,51 (0.03)<br>1,47 (0.03)
PAK4	p21(CDKN1A)-activated kinase 4	1,98 (0.02<br>1,88 (0.01))
RHOF	Ras homolog gene family, member F (in filopodia)	1,92 (0.03)<br>1,59 (8.24 x 10^{-3})
PAK7	p21(CDKN1A)-activated kinase 7	1,88 (7.41 x 10^{-4})<br>1,41 (4.55 x 10^{-4})
ARHGAP1	Rho GTPase activating protein 1	1,85 (0.04)
BCR	breakpoint cluster region	1,80 (0.01)
PDLIM7	PDZ and LIM domain 7 (enigma)	1,80 (0.01)<br>1.06 (3,24 x 10^{-3})
PIK3CB	phosphoinositide-3-kinase, catalytic, beta polypeptide	1,74 (6.64 x 10^{-3})
STAT1	signal transducer and activator of transcription 1, 91kDa	1,72 (1.21 x 10^{-4})
PIK3C2B	phosphoinositide-3-kinase, class 2, beta polypeptide	1,6 (6.94 x 10^{-3})
PAK2	p21 (CDKN1A)-activated kinase 2	1,56 (5.78 x 10^{-3})<br>1,55 (1.20 x 10^{-3})
BIRC5	baculoviral IAP repeat-containing 5 (survivin)	1,54 (7.37 x 10^{-3})<br>1,54 (2.48 x 10^{-3})<br>1,50 (0.03)
DVL2	dishevelled, dsh homolog 2 (Drosophila)	1,54 (0.01)<br>1,32 (0.03)

BRAF	v-raf murine sarcoma viral oncogene homolog B1	1,53 (0.01)
MAP3K1	mitogen-activated protein kinase kinase kinase 1	1,53 (0.03)
ABR	active BCR-related gene	1,47 (0.03)
MAPKAPK2	mitogen-activated protein kinase-activated protein kinase 2	1,45 (1.83×10^{-3})
DOK1	docking protein 1, 62kDa (downstream of tyrosine kinase 1)	1,44 (0.01)
MAPK12	mitogen-activated protein kinase 12	1,41 (7.63×10^{-3})
NRAS	neuroblastoma RAS viral (v-ras) oncogene homolog	1,40 (0.02)
AKT1	v-akt murine thymoma viral oncogene homolog 1	1,38 (0.02)
JUN	jun oncogene	1,38 (0.01)<br>1.28 (0.02)
WNT5A	wingless-type MMTV integration site family, member 5A	1,29 (4.95×10^{-3})
PLCG1	phospholipase C, gamma 1	1,28 (0.03)
TALDO1	transaldolase 1	1,19 (0.04)
HSH2D	hematopoietic SH2 domain containing	0,93 (7.81×10^{-3})
LOC647836 /// LOC649279 /// WNT7B	wingless-type MMTV integration site family, member 7B /// similar to wingless-type MMTV integration site family, member 7B precursor	0,89 (0.03)
MAPK14	mitogen-activated protein kinase 14	0,82 (0.04)
KRAS	v-Ki-ras2 Kirsten rat sarcoma viral oncogene homolog	0,80 (0.03)
EPHA8	EPH receptor A8	0,77 (0.01)
GSK3A	glycogen synthase kinase 3 alpha	0,75 (0.04)
CTNNB1		0,72 (7.19×10^{-4})

	catenin (cadherin-associated protein), beta 1, 88kDa	
WNT5B	wingless-type MMTV integration site family, member 5B	0,71 (0.04)
AXIN2	axin 2 (conductin, axil)	0,70 (0.02)
MAPK1	mitogen-activated protein kinase 1	0,69 (2.27×10^{-3})
MAPK8	mitogen-activated protein kinase 8	0,68 (0.01)<br>0.66 (0.01)
HRAS	v-Ha-ras Harvey rat sarcoma viral oncogene homolog	0,65 (0.04)
JAG2	jagged 2	0,62 (0.02)
PRKCE	protein kinase C, epsilon	0,61 (1.06×10^{-3})
PTPN11	protein tyrosine phosphatase, non-receptor type 11 (Noonan syndrome 1)	0,61 (0.02)
PIK3R1	phosphoinositide-3-kinase, regulatory subunit 1 (p85 alpha)	0,59 (0.01)<br>0.48 (0.03)<br>0.45 (9.66×10^{-3})
EPHA5	EPH receptor A5	0,58 (0.02)
GRB10	growth factor receptor-bound protein 10	0,58 (0.01)
PAK3	p21 (CDKN1A)-activated kinase 3	0,56 (0.01)
FRS2	fibroblast growth factor receptor substrate 2	0,49 (9.11×10^{-3})
SOS1	son of sevenless homolog 1 (Drosophila)	0,47 (2.37×10^{-3})<br>0.25 (2.42×10^{-3})
FRZB	frizzled-related protein	0,45 (8.80×10^{-5})<br>0.40 (2.46×10^{-5})
EPHA7	EPH receptor A7	0,44 (2.80×10^{-3})<br>0.30 (1.50×10^{-3})
FZD8	frizzled homolog 8 (Drosophila)	0,41 (4.54×10^{-4})
MAPK10	mitogen-activated protein kinase 10	0,41 (5.24×10^{-4})
SPHK1		0,41 (2.10×10^{-4})

	sphingosine kinase 1	
PRKCA	protein kinase C, alpha	0,09 (1.75 x 10^{-6})
