# Supplementary material for: Chemoresistance acquisition induces a global shift of expression of aniogenesis-associated genes and increased pro-angogenic activity in neuroblastoma cells
Source: Mol Cancer. 2009 Sep 29;8:80. doi: 10.1186/1476-4598-8-80 (PMC2761864; doi:10.1186/1476-4598-8-80)
Supplement: Additional file 7 — Endothelial cell tube formation induced by supernatants of neuroblastoma cells. Quantification of endothelial cell tube formation induced by supernatants of neuroblastoma cells. [file 1476-4598-8-80-S7.PDF]

Additional file 7

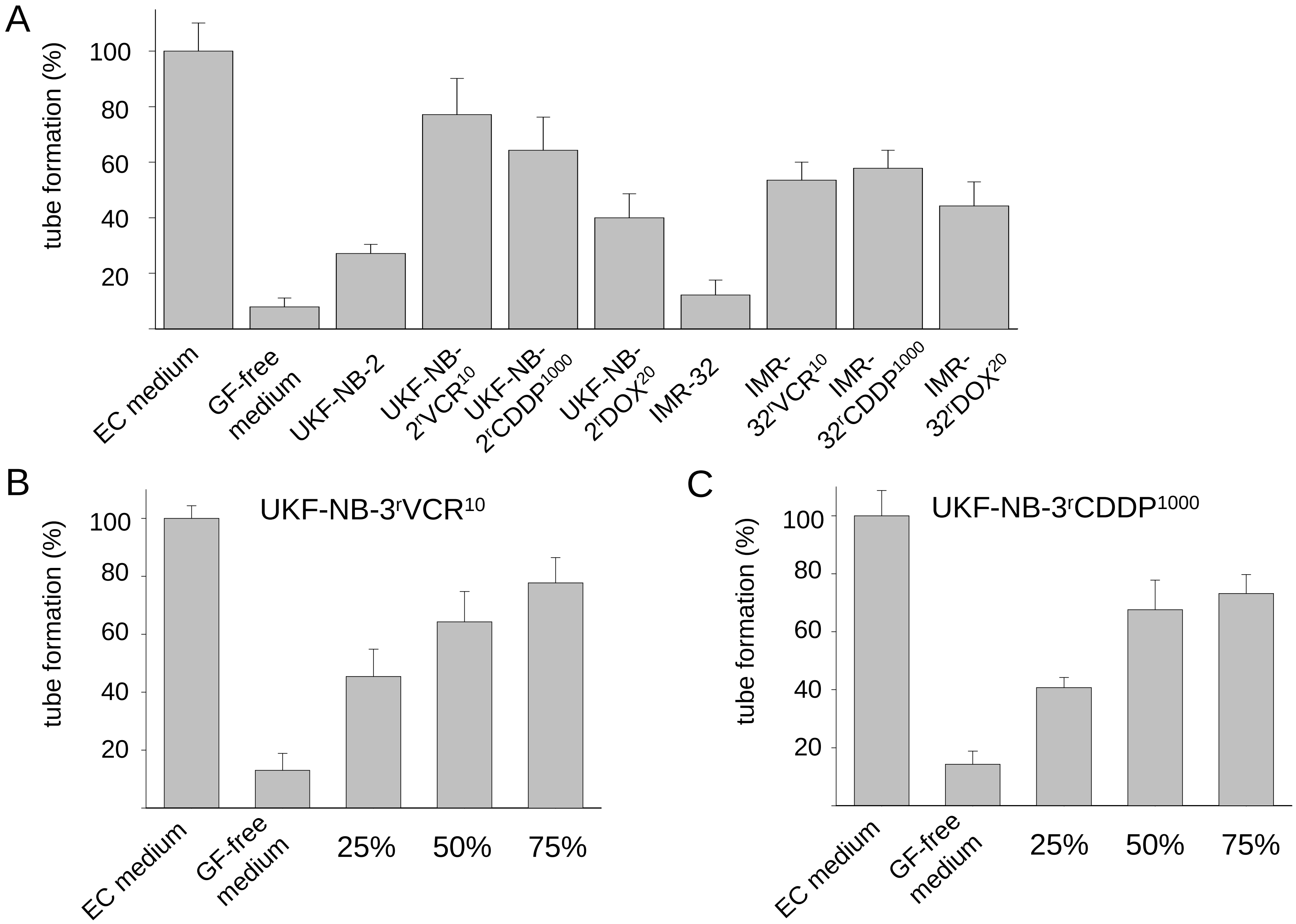

Additional file 7. A) Quantification of tube formation by counting of branching points of HUVEC incubated with 1:1 mixtures of IMDM and supernatants of chemosensitive neuroblastoma cell lines (UKF-NB-2, IMR-32) and the corresponding chemoresistant sub-lines (UKF-NB-2^{r}VCR^{10}, UKF-NB-2^{r}CDDP^{1000}, UKF-NB-2^{r}DOX^{20}, IMR-32^{r}VCR^{10}, IMR-32^{r}CDDP^{1000}, IMR-32^{r}DOX^{10}) + 10% FCS 16h after seeding on extracellular matrix in comparison to endothelial cell medium (EC medium, IMDM + 15% FCS + 5% pooled human serum + bFGF 2.5 ng/ml) and growth factor-free medium (GF-free medium, IMDM + 10% FCS). $P < 0.05$ relative to endothelial cells incubated with the supernatants of the corresponding parental cells B) Influence of different ratios of IMDM and supernatants of UKF-NB-3^{r}VCR^{10} cells (25%, 50%, or 75% UKF-NB-3^{r}VCR^{10} supernatant) + 10% FCS on tube formation 16h after seeding on extracellular matrix in comparison to EC medium or GF-free medium. C) Influence of different ratios of IMDM + 10% FCS and supernatants of UKF-NB-3^{r}CDDP^{1000} cells (25%, 50%, or 75% UKF-NB-3^{r}CDDP^{1000} supernatant) on tube formation 16h after seeding on extracellular matrix in comparison to EC medium or GF-free medium.
