# Supplementary material for: Chemoresistance acquisition induces a global shift of expression of aniogenesis-associated genes and increased pro-angogenic activity in neuroblastoma cells
Source: Mol Cancer. 2009 Sep 29;8:80. doi: 10.1186/1476-4598-8-80 (PMC2761864; doi:10.1186/1476-4598-8-80)
Supplement: Additional file 8 — Densitometric analysis of Western blot analyses. Densitometric analysis of Western blot analyses investigating expression of ERK 1/2, phosphorylated ERK 1/2 (pERK 1/2), Akt, Akt phosphorylated at Ser473 (pAkt Ser473), or Akt phosphorylated at Thr308 (pAkt Thr308) in endothelial cells incubated supernatants of UKF-NB-3, UKF-NB-3rVCR10, UKF-NB-3rCDDP1000, or UKF-NB-3rDOX20 cells. [file 1476-4598-8-80-S8.PDF]

Additional file 8

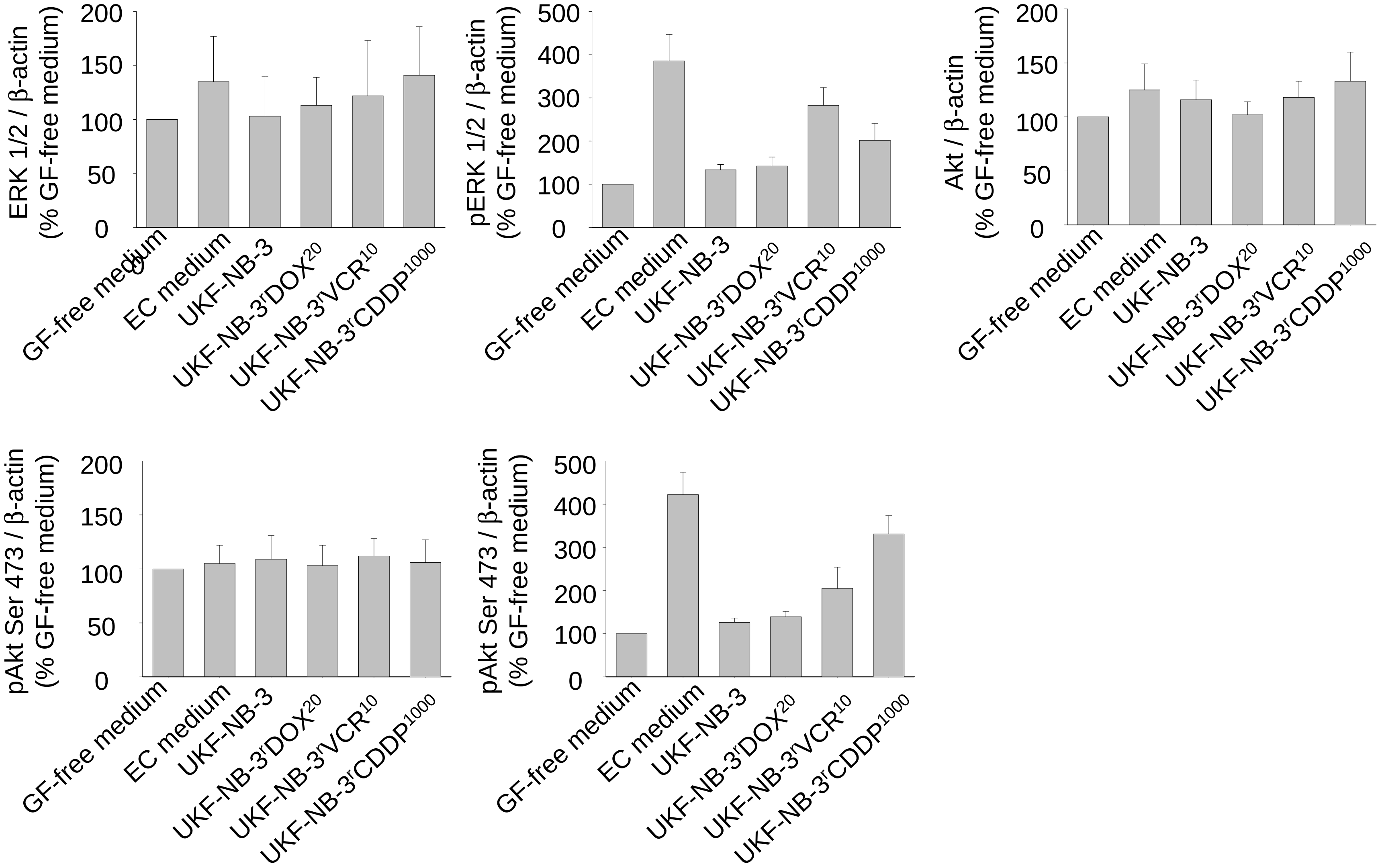

Additional file 8. Densitometric analysis of Western blot analyses investigating expression of ERK 1/2, phosphorylated ERK 1/2 (pERK 1/2), Akt, Akt phosphorylated at Ser473 (pAkt Ser473), or Akt phosphorylated at Thr308 (pAkt Thr308) in endothelial cells incubated with 1:1 mixtures of IMDM and supernatants of UKF-NB-3, UKF-NB-$3^{r}VCR^{10}$, UKF-NB-$3^{r}CDDP^{1000}$, or UKF-NB-$3^{r}DOX^{20}$ cells + 10% FCS in comparison to EC medium and GF-free medium for 24 h. β-actin was used as loading control.
