# Supplementary material for: Chemoresistance acquisition induces a global shift of expression of aniogenesis-associated genes and increased pro-angogenic activity in neuroblastoma cells
Source: Mol Cancer. 2009 Sep 29;8:80. doi: 10.1186/1476-4598-8-80 (PMC2761864; doi:10.1186/1476-4598-8-80)
Supplement: Additional file 9 — Pro-angiogenic factors in the supernatants of neuroblastoma cell lines. Concentrations of selected pro-angiogenic factors in the supernatants of neuroblastoma cell lines. [file 1476-4598-8-80-S9.PDF]

Additional file 9. Concentrations of selected pro-angiogenic factors in the supernatants of neuroblastoma cell lines (detected by BD Cytometric Bead Array, Becton Dickinson, Heidelberg, Germany, following the manufacturer's instructions).

	Concentrations (pg/ml) of				
**Cell line**	**IL-8**	**angiogenin**	**bFGF**	**TNF-α**	**VEGF**
UKF-NB-3	n.d.*	142	46	n.d.	410
UKF-NB-3^{r}VCR^{10}	n.d.	n.d.	47	n.d.	860
UKF-NB-3^{r}DOX^{20}	n.d.	n.d.	n.d.	n.d.	520
UKF-NB-3^{r}CDDP^{1000}	n.d.	316	47	n.d.	1550
UKF-NB-2	n.d.	n.d.	315	n.d.	> 5000
UKF-NB-2^{r}VCR^{10}	n.d.	n.d.	n.d.	n.d.	n.d.
UKF-NB-2^{r}DOX^{20}	n.d.	n.d.	n.d.	n.d.	955
UKF-NB-2^{r}CDDP^{1000}	n.d.	n.d.	138	n.d.	1490
IMR-32	n.d.	n.d.	n.d.	n.d.	1370
IMR-32^{r}VCR^{10}	n.d.	n.d.	n.d.	n.d.	985
IMR-32^{r}DOX^{20}	n.d.	n.d.	n.d.	n.d.	3250
IMR-32^{r}CDDP^{1000}	n.d.	n.d.	n.d.	n.d.	> 5000
UKF-NB-4	n.d.	n.d.	n.d.	n.d.	> 5000
Be(2)-C	n.d.	1300	n.d.	n.d.	> 5000

* n.d. = not detectable
