# Supplementary material for: Chemoresistance acquisition induces a global shift of expression of aniogenesis-associated genes and increased pro-angogenic activity in neuroblastoma cells
Source: Mol Cancer. 2009 Sep 29;8:80. doi: 10.1186/1476-4598-8-80 (PMC2761864; doi:10.1186/1476-4598-8-80)
Supplement: Additional file 10 — Expression of stemness genes. Comparison of expression of selected stemness genes in chemoresistant or chemosensitive neuroblastoma cells. [file 1476-4598-8-80-S10.PDF]

Additional file 10. Expression of selected stemness genes (detected by gene microarray analysis).

Gene ID	Encoded protein	relative expression UKF-NB-$3^{r}VCR^{10}$ versus UKF-NB-3 (multiple-testing corrected p-value based on three biological replicates)	relative expression UKF-NB-$3^{r}CDDP^{1000}$ versus UKF-NB-3 (multiple-testing corrected p-value based on three biological replicates)
NANOG	Nanog	no signal[1]<br>0.364 (0.094)	0.528 (0.499)<br>0.652 (0.655)
POU5F1	Oct4	no signal<br>0.403 (0.156)<br>no signal	0.489 (0.263)<br>0.223 (0.049)<br>0.724 (0.186)
PCGF4	Bmi-1	1.224 (0.606)<br>0.670 (0.038)	0.647 (0.490)<br>0.491 (0.300)

[1] no expression detected
