# Supplementary material for: Chemoresistance acquisition induces a global shift of expression of aniogenesis-associated genes and increased pro-angogenic activity in neuroblastoma cells
Source: Mol Cancer. 2009 Sep 29;8:80. doi: 10.1186/1476-4598-8-80 (PMC2761864; doi:10.1186/1476-4598-8-80)
Supplement: Additional file 11 — Hierarchical cluster analysis of expresson of angiogenesis-associated genes. Hierarchical cluster analysis and heatmap showing expression of angiogenesis-associated genes in UKF-NB-2, UKF-NB-2rVCR10, or UKF-NB-2rCDDP1000 cells. [file 1476-4598-8-80-S11.PDF]

Additional file 11

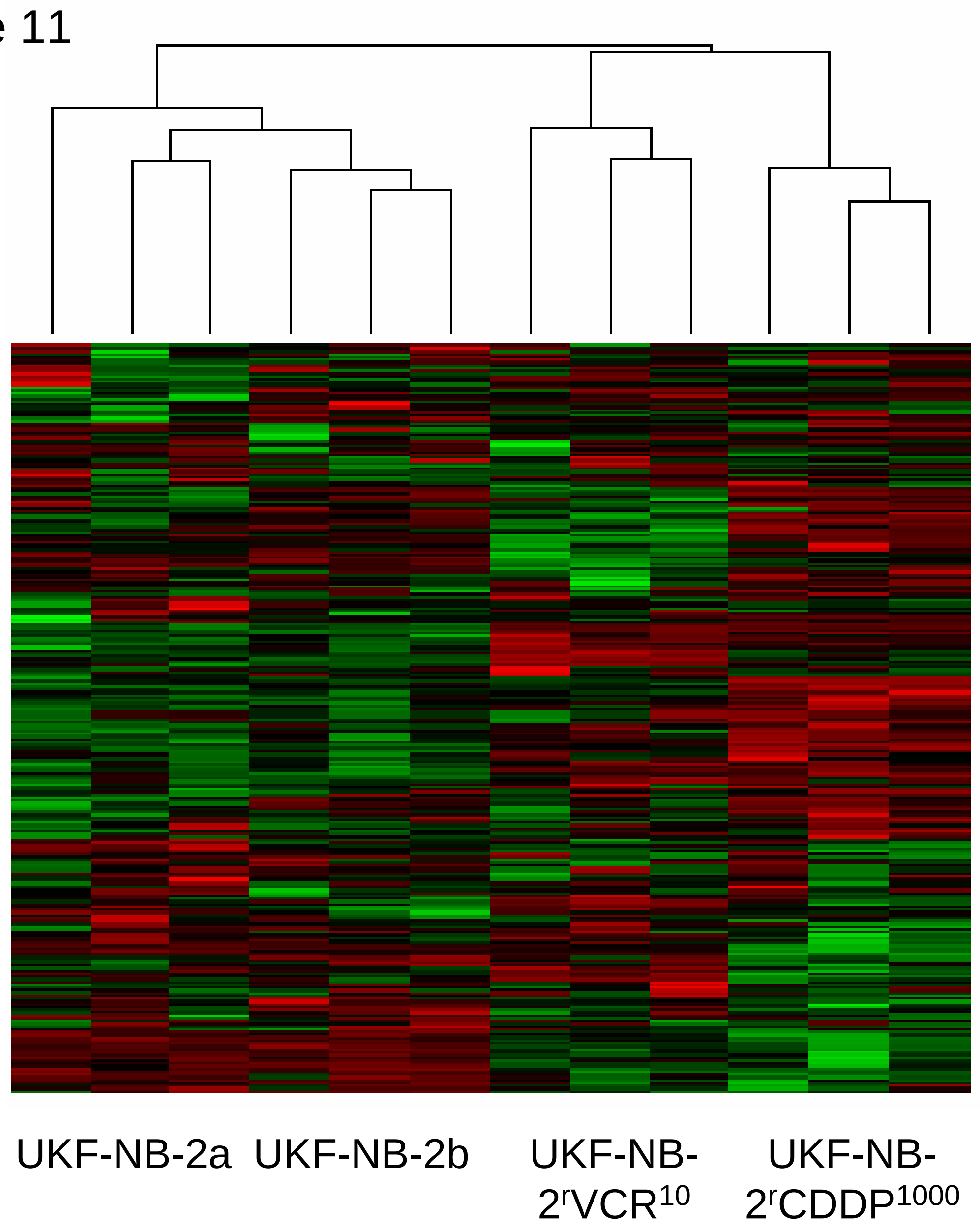

Additional file 11. Hierarchical cluster analysis and heatmap showing expression of angiogenesis-associated genes (taken from PANTHER pathway) in UKF-NB-2, UKF-NB-2^{r}VCR^{10}, or UKF-NB-2^{r}CDDP^{1000} cells. Data were merged from two independent experiments each comparing UKF-NB-2 cells with a chemoresistant sub-line. UKF-NB-2a was analysed together with UKF-NB-2^{r}VCR^{10}. UKF-NB-2b was analysed together with UKF-NB-2^{r}CDDP^{1000}.
