# Supplementary material for: Chemoresistance acquisition induces a global shift of expression of aniogenesis-associated genes and increased pro-angogenic activity in neuroblastoma cells
Source: Mol Cancer. 2009 Sep 29;8:80. doi: 10.1186/1476-4598-8-80 (PMC2761864; doi:10.1186/1476-4598-8-80)
Supplement: Additional file 12 — Hierarchical cluster analysis of expresson of angiogenesis-associated genes. Hierarchical cluster analysis and heatmap showing expression of angiogenesis-associated genes in IMR-32 or IMR-32rVCR10 cells. [file 1476-4598-8-80-S12.PDF]

Additional file 12

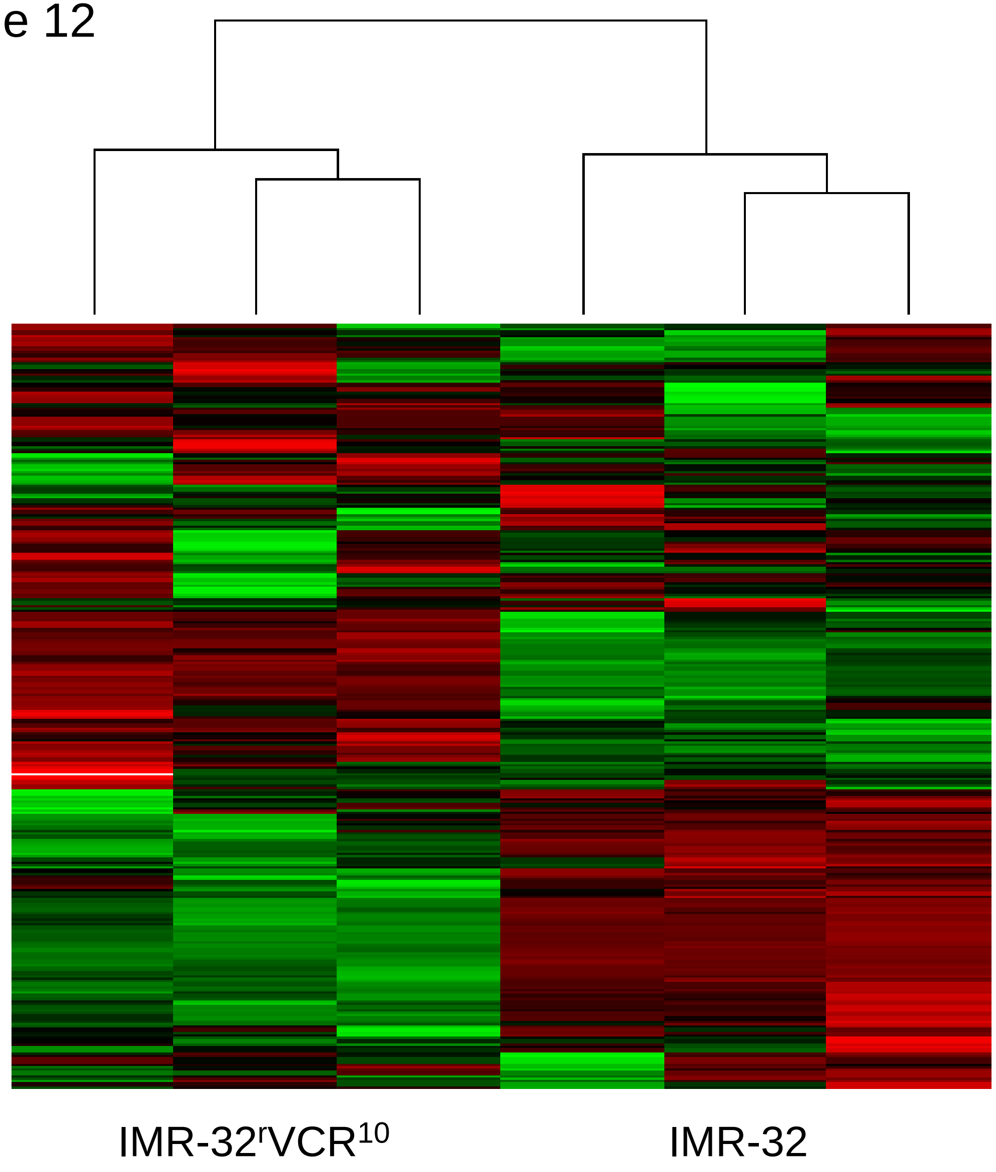

Additional file 12. Hierarchical cluster analysis and heatmap showing expression of angiogenesis-associated genes (taken from PANTHER pathway) in IMR-32 or IMR-32^{r}VCR^{10} cells.
