# Supplementary material for: Chemoresistance acquisition induces a global shift of expression of aniogenesis-associated genes and increased pro-angogenic activity in neuroblastoma cells
Source: Mol Cancer. 2009 Sep 29;8:80. doi: 10.1186/1476-4598-8-80 (PMC2761864; doi:10.1186/1476-4598-8-80)
Supplement: Additional file 13 — Bioinformatic pathway analysis in neuroblastoma patients' tumours. Signalling pathways most strongly differentially regulated between N-myc amplified neuroblastoma tissues from patients with favourable outcome or poor outcome. [file 1476-4598-8-80-S13.PDF]

Additional file 13. The top four PANTHER signalling pathways (taken from www.pantherdb.org) most strongly differentially regulated between N-myc amplified neuroblastoma tissues from patients with favourable outcome (event-free survival) or poor outcome (death or relapse of disease). Neuroblastoma gene expression data was obtained from ArrayExpress (www.ebi.ac.uk/arrayexpress; accession E-TABM-38; see Oberthuer et al., J Clin Oncol 2006;24:5070-8).

**PANTHER signalling pathway**	**p-value**
**1. Angiogenesis**	**2.71×10^{-14}**
2. Inflammation mediated by chemokine and cytokine signalling pathway	3.03×10^{-12}
3. PDGF signalling pathway	2.87×10^{-9}
4. Ras pathway	1.30×10^{-8}
