# Supplementary material for: Chemoresistance acquisition induces a global shift of expression of aniogenesis-associated genes and increased pro-angogenic activity in neuroblastoma cells
Source: Mol Cancer. 2009 Sep 29;8:80. doi: 10.1186/1476-4598-8-80 (PMC2761864; doi:10.1186/1476-4598-8-80)
Supplement: Additional file 14 — Bioinformatic pathway analysis in neuroblastoma patients' tumours. Signalling pathways most strongly differentially regulated between non-N-myc amplified neuroblastoma tissues from patients with favourable outcome or poor outcome. [file 1476-4598-8-80-S14.PDF]

Additional file 14. The top four PANTHER signalling pathways (taken from www.pantherdb.org) most strongly differentially regulated between non-N-myc amplified neuroblastoma tissues from patients with favourable outcome (no relapse) or poor outcome (relapse of disease). Neuroblastoma gene expression data was obtained from Gene Expression Omnibus (www.ncbi.nlm.nih.gov/geo/; accession number GSE3446; Asgharzadeh et al., J Natl Cancer Inst 2006;98:1193-203).

**PANTHER signalling pathway**	**p-value**
1. Wnt signalling pathway	2.80×10^{-16}
2. Huntington disease	6.53×10^{-11}
**3. Angiogenesis**	**7.68×10^{-11}**
4. PDGF signalling pathway	3.55×10^{-9}
